# *In Vivo* Development of Aztreonam Resistance in Meropenem-Resistant Pseudomonas aeruginosa Owing to Overexpression of the *bla*_PDC-16_

**DOI:** 10.1128/spectrum.03080-22

**Published:** 2023-04-18

**Authors:** Li Ding, Yue Sun, Yizhuo Zhang, Siquan Shen, Fupin Hu

**Affiliations:** a Institute of Antibiotics, Huashan Hospital, Fudan University, Shanghai, People’s Republic of China; b Key Laboratory of Clinical Pharmacology of Antibiotics, Ministry of Health, Shanghai, People’s Republic of China; Innovations Therapeutiques et Resistances

**Keywords:** *Pseudomonas aeruginosa*, *bla*_IMP-45_, *bla*_PDC-16_

## Abstract

The rapid acquisition of antibiotic resistance of Pseudomonas aeruginosa has been a complex problem in clinics. Two meropenem-resistant P. aeruginosa isolates were collected from the same patient on May 24, 2021, and June 4, 2021, respectively. The first was susceptible to aztreonam, while the second displayed resistance. This study aimed to identify the genetic differences between two P. aeruginosa isolates and uncover alterations formed by the within-host bacterial evolution leading to aztreonam resistance during therapy. Strains were subjected to antimicrobial susceptibility testing using the broth microdilution method. Genomic DNAs were obtained to identify their genetic differences. The relative mRNA levels of β-lactam-resistance genes were determined by real-time PCR. Both isolates belonged to ST 773 high-risk clones with the same antibiotic resistance genes, eliminating the possibility of horizontally obtaining resistance genes. Reverse transcription (RT)-PCR results showed that the *bla*_PDC-16_ mRNA level in the second one was about 1,500 times higher than that in the first one. When 3-aminophenyl boronic acid was added, the second strain recovered its susceptibility to aztreonam, which confirmed that the overexpression of *bla*_PDC-16_ was the main reason for the isolate’s resistance to aztreonam. Compared to the first strain, the second showed a single amino acid substitution in AmpR located upstream of *bla*_PDC-16_, which may contribute to the upregulation of *bla*_PDC-16_ and lead to aztreonam resistance. *AmpR* plays an essential role in regulating antibiotic resistance in P. aeruginosa, and there is a need to be alert to clinical treatment failures associated with mutations in *ampR*.

**IMPORTANCE** Pseudomonas aeruginosa is notorious for being highly resistant to antimicrobial agents. In this study, two P. aeruginosa strains isolated from the same patient with different susceptibility to aztreonam were used to illustrate the within-host resistance evolution process of P. aeruginosa. Both isolates, which belonged to a ST773 high-risk clone, had the same β-lactam resistance genes (*bla*_PDC-16_, *bla*_IMP-45_, *bla*_OXA-1_, and *bla*_OXA-395_), which means the second isolate might have been derived from the first isolate by gaining aztreonam resistance via mutations associated with aztreonam resistance relative genes. Subsequently, we found that mutation in *ampR* may be the cause of aztreonam resistance in the second isolate. Mutation in *ampR* leads to its loss of control over *bla*_PDC-16_, allowing overexpression of *bla*_PDC-16_ and further resistance to aztreonam. This study revealed that *ampR* plays an essential role in regulating antibiotic resistance in P. aeruginosa. There is a need to be alert to clinical treatment failures associated with mutations in *ampR*.

## INTRODUCTION

Carbapenem-resistant Pseudomonas aeruginosa (CRPA) is a common pathogen in nosocomial infections, such as ventilator-associated pneumonia, postoperative infections, wound infections, and potentially life-threatening bloodstream infections (1, 2). Patients with severe burn wounds are highly susceptible to opportunistic infections due to the loss of the protective skin barrier and numerous injury-induced immune alterations that impair the ability to control the spread of disease (3, 4). According to China Antimicrobial Surveillance Network (CHINET) data in China 2021 (http://www.chinets.com/Data/AntibioticDrugFast), CRPA isolated from hospitals has reached 24.7%, making treatment challenging. Moreover, the growing prevalence of nosocomial infections caused by CRPA is associated with increased morbidity and mortality (5, 6).

The hyperproduction of the intrinsic inducible cephalosporinase AmpC is the primary mechanism used by P. aeruginosa to cope with β-lactams (7, 8). Meanwhile, AmpC hyperproduction is intimately linked to the peptidoglycan recycling pathway, regulated by an *AmpG-AmpD-NagZ-AmpR* regulatory mechanism in Gram-negative bacteria (9). *AmpR*, a LysR-type transcriptional regulator usually located upstream of the β-lactamase gene, was classified as regulating the expression of the chromosomally encoded β-lactamase gene via an *AmpG-AmpD-NagZ-AmpR* regulatory mechanism (10). The emergence of a D135N AmpR mutant resistant to ceftolozane-tazobactam *in vitro* was reported during evolution experiments performed with a hypermutable PAO1 strain exposed to ceftazidime (11, 12). A similar mutant expressing fully derepressed expression levels of the Pseudomonas-derived cephalosporinase (PDC) β-lactamase was selected under ceftazidime treatment from a cystic fibrosis strain in a murine model of chronic lung infection (11, 13). In addition, several extensively drug-resistant strains from various geographical origins have also been detected to carry a D135N mutation, emphasizing the importance of AmpR in β-lactam resistance acquisition (14). However, the exact evolution processes of a susceptible strain to a resistant clone are unknown. This study aimed to illustrate the D135G substitution of AmpR, leading to the switch from susceptibility to the resistance of P. aeruginosa harboring *bla*_IMP-45_ to aztreonam through a clinical case, highlighting the importance of the global transcriptional regulator ampR.

## RESULTS

### Antimicrobial susceptibility and carbapenemase detection of P. aeruginosa HS90 and P. aeruginosa HS110.

P. aeruginosa HS90 and P. aeruginosa HS110 had similar antimicrobial susceptibility patterns, except for aztreonam (Table 1). They were all resistant to meropenem, ceftazidime, cefepime, piperacillin-tazobactam, ceftazidime-avibactam, and amikacin, while they also displayed susceptibility to imipenem, cefepime-zidebactam, polymyxin B, and cefiderocol. In addition, the MICs of fosfomycin were both 32 mg/liter, but the Clinical and Laboratory Standards Institute (CLSI) currently does not provide susceptibility breakpoints for fosfomycin specific to P. aeruginosa. Interestingly, the subsequent isolate P. aeruginosa HS110 was resistant to aztreonam (MIC = 64 mg/liter), while the first strain P. aeruginosa HS90 was susceptible to aztreonam (MIC = 4 mg/liter). Additionally, both P. aeruginosa HS90 and P. aeruginosa HS110 were *bla*_IMP_-positive by using NG test Carba-5.

**TABLE 1:** Antimicrobial susceptibility and efflux pump inhibitory assays of P. aeruginosa HS90 and P. aeruginosa HS110^*a*^

Antimicrobial agent	MICs (mg/liter) of P. aeruginosa HS90			MICs (mg/liter) of P. aeruginosa HS110		
	MIC	+CCCP (25 mg/liter)	+PAβN (25 mg/liter)	MIC	+CCCP (25 mg/liter)	+PAβN (25 mg/liter)
Aztreonam	4 S	2 S	2 S	64 R	32 R	32 R
Ceftazidime	512 R	256 R	256 R	256 R	256 R	256 R
Cefepime	128 R	64 R	128 R	128 R	64 R	128 R
Cefiderocol	0.5 S	ND	ND	0.5 S	ND	ND
Meropenem	32 R	16 R	64 R	32 R	16 R	64 R
Imipenem	4 I	1 S	4 I	2 S	1 S	4 I
Piperacillin-tazobactam	128 R	64 R	128 R	256 R	128 R	256 R
Cefoperazone-sulbactam	>128 R	128 R	>128 R	>128 R	128 R	>128 R
Ceftazidime-avibactam	512 R	256 R	256 R	256 R	256 R	256 R
Cefepime-zidebactam	4 S	4 S	4 S	8 S	8 S	4 S
Levofloxacin	16 R	8 R	1 S	16 R	1 S	1 S
Ciprofloxacin	8 R	4 R	2 R	8 R	4 R	2 R
Fosfomycin	32	ND	ND	32	ND	ND
Polymyxin B	1 S	0.5 S	0.25 S	0.5 S	0.5 S	0.25 S
Amikacin	>128 R	>128 R	>128 R	>128 R	>128 R	>128 R

a R, resistant; S, susceptible; ND, not determined.

### WGS comparison of P. aeruginosa HS90 and P. aeruginosa HS110.

According to whole-genome sequencing (WGS) data, both P. aeruginosa HS90 and P. aeruginosa HS110 belonged to ST 773 and O11 serotype and carried the same β-lactam resistance genes (*bla*_PDC-16_, *bla*_IMP-45_, *bla*_OXA-1_, and *bla*_OXA-395_), quinolone resistance gene *qnrVC1*, and aminoglycoside resistance genes (*armA*, *aph(3′)-Ia*, and *aac(6′)-Ib-cr*), etc. Compared to P. aeruginosa HS90, no extra β-lactam resistance gene was found in P. aeruginosa HS110 (Table 2).

**TABLE 2:** Resistance genes found in P. aeruginosa HS90 and P. aeruginosa HS110

Antimicrobial class	P. aeruginosa HS90/P. aeruginosa HS110 genes
β-Lactams	*bla*_PDC-16,_ *bla*_IMP-45_, *bla*_OXA-1_, *bla*_OXA-395_
Quinolone	*qnrVC1*, *aac(6′)-Ib-cr*, *crpP*
Aminoglycoside	*armA*, *aph(3′)-Iib*, *aac(6′)-Ib3, aac(6′)-Ib-cr, aph(3′)-Ia*
Fosfomycin	*fosA*
Sulfonamide	*sul1*
Trimethoprim	*dfrA22*

### Expression of the β-lactam resistance genes and *mexAB-OprM* efflux pump genes of P. aeruginosa HS90 and P. aeruginosa HS110.

**FIG 1:**
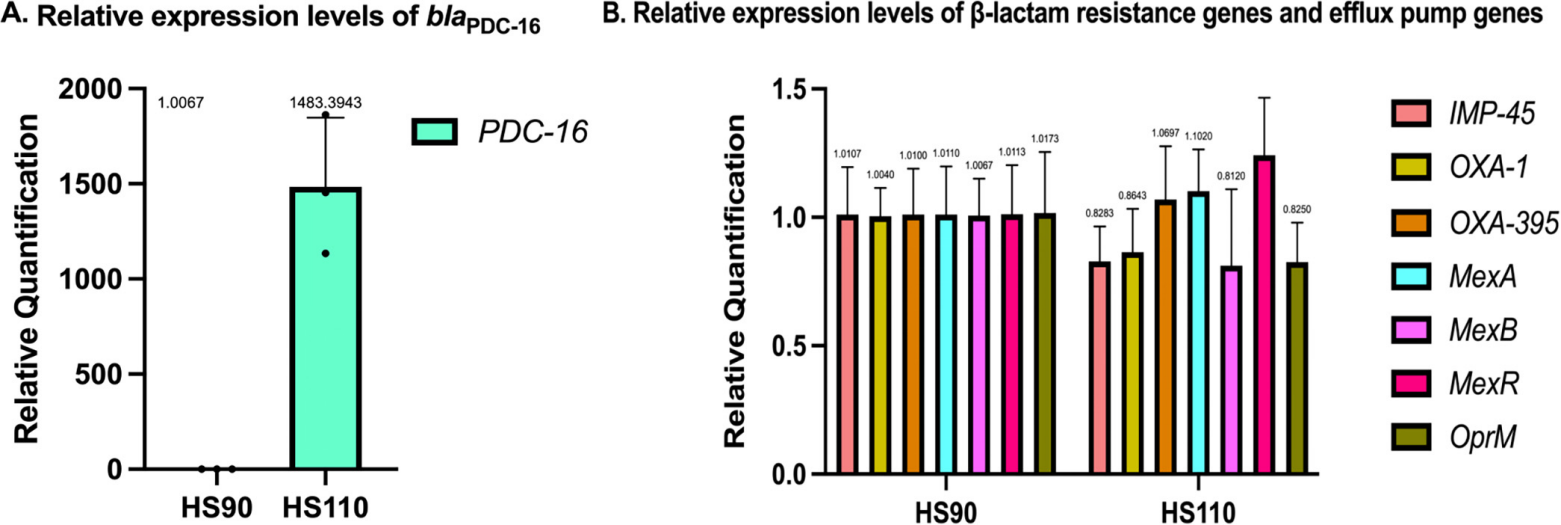
(A) Relative mRNA expression of *bla*_PDC-16_ in *P. aeruginosa* HS90 and *P. aeruginosa* HS110. (B) Relative mRNA expression of *bla*_OXA-1_, *bla*_OXA-395_, *bla*_IMP-45_, and *mexAB-OprM* efflux pump in *P. aeruginosa* HS90 and *P. aeruginosa* HS110.

The β-lactam resistance genes (*bla*_PDC-16_, *bla*_IMP-45_, *bla*_OXA-1_, *bla*_OXA-395_) and efflux pump genes (including *MexA*, *MexB*, *MexR*, and *OprM*) expression of the two isolates was compared at the mRNA levels. Interestingly, the *bla*_PDC-16_ mRNA level in P. aeruginosa HS110 was about 1,500 times higher than in P. aeruginosa HS90. Meanwhile, the other β-lactam resistance genes (*bla*_IMP-45_, *bla*_OXA-1_, and *bla*_OXA-395_) mRNA levels in P. aeruginosa HS110 were not obviously different than that in P. aeruginosa HS90. In addition, the mRNA levels of the efflux pump genes (including *MexA*, *MexB*, *MexR*, and *OprM*) were not very different between P. aeruginosa HS90 and P. aeruginosa HS110 (Fig. 1).

When the nonspecific efflux pump inhibitor carbonyl cyanide *m*-chlorophenylhydrazone (CCCP) and the AcrAB-TolC pump inhibitor phenylalanine-arginine β-naphthylamide (PAβN) were added, the MIC of aztreonam in the two strains had a 2-fold change. When adding CCCP or PAβN, the aztreonam MIC of P. aeruginosa HS90 decreased from 4 to 2 mg/liter (Table 1). When adding CCCP or PAβN, the aztreonam MIC of P. aeruginosa HS110 decreased from 64 to 32 mg/liter. Based on the above results, we speculate that efflux pumps do not play the most important role in aztreonam resistance.

**FIG 2:**
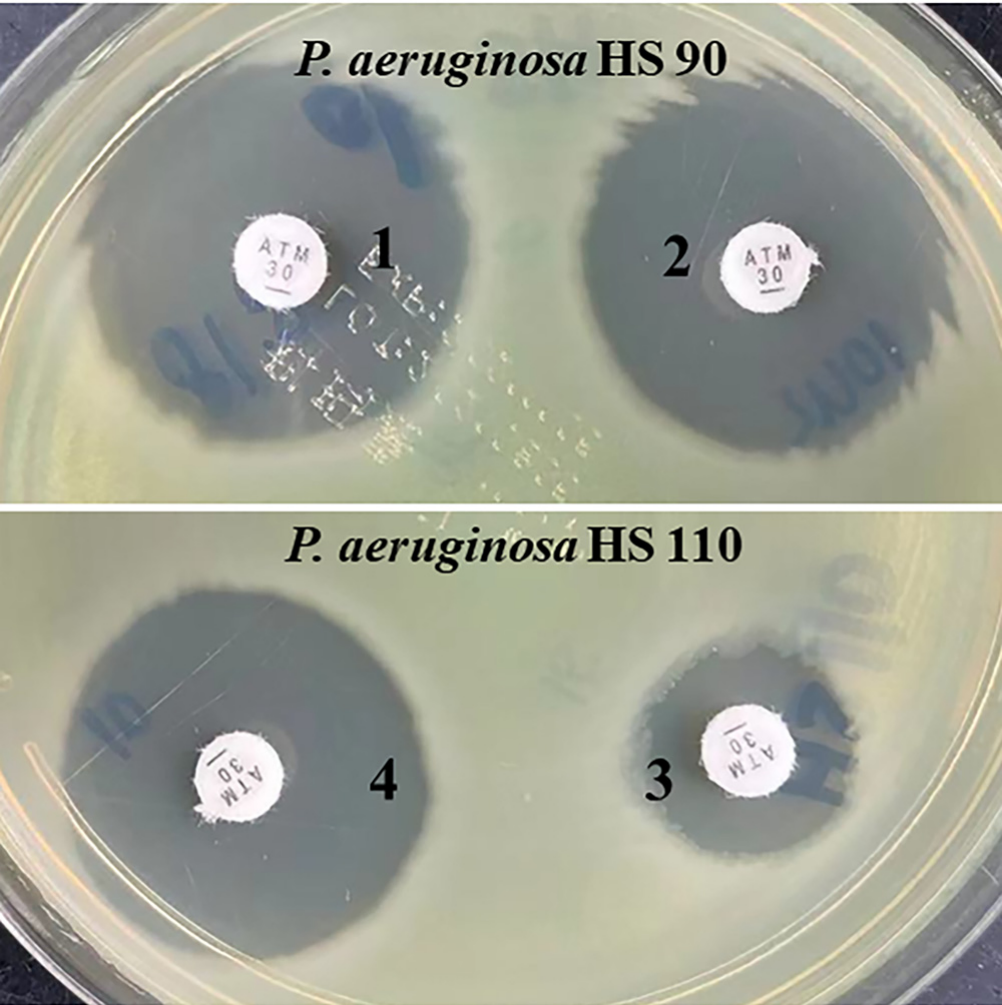
The inhibitory zone diameter around aztreonam disc alone or plus 3-aminophenyl boronic acid (APB) in P. aeruginosa HS90 and P. aeruginosa HS110. Zone 1, aztreonam (30 μg), 26 mm; zone 2, aztreonam (30 μg) plus APB (300 μg/disc), 26 mm; zone 3, aztreonam (30 μg), 15 mm; zone 4, aztreonam (30 μg) plus APB (300 μg/disc), 26 mm.

Subsequently, we compared the aztreonam susceptibility using aztreonam disc and aztreonam disc plus 3-aminophenyl boronic acid (APB) (300 μg/disc). The results showed that P. aeruginosa HS110 had restored aztreonam susceptibility *in vitro* when adding APB. Meanwhile, the inhibitory zone diameter around aztreonam plus APB of P. aeruginosa HS90 was not obviously different from aztreonam alone (Fig. 2).

### Change in aztreonam susceptibility by the mutation of AmpR D135G.

**FIG 3:**
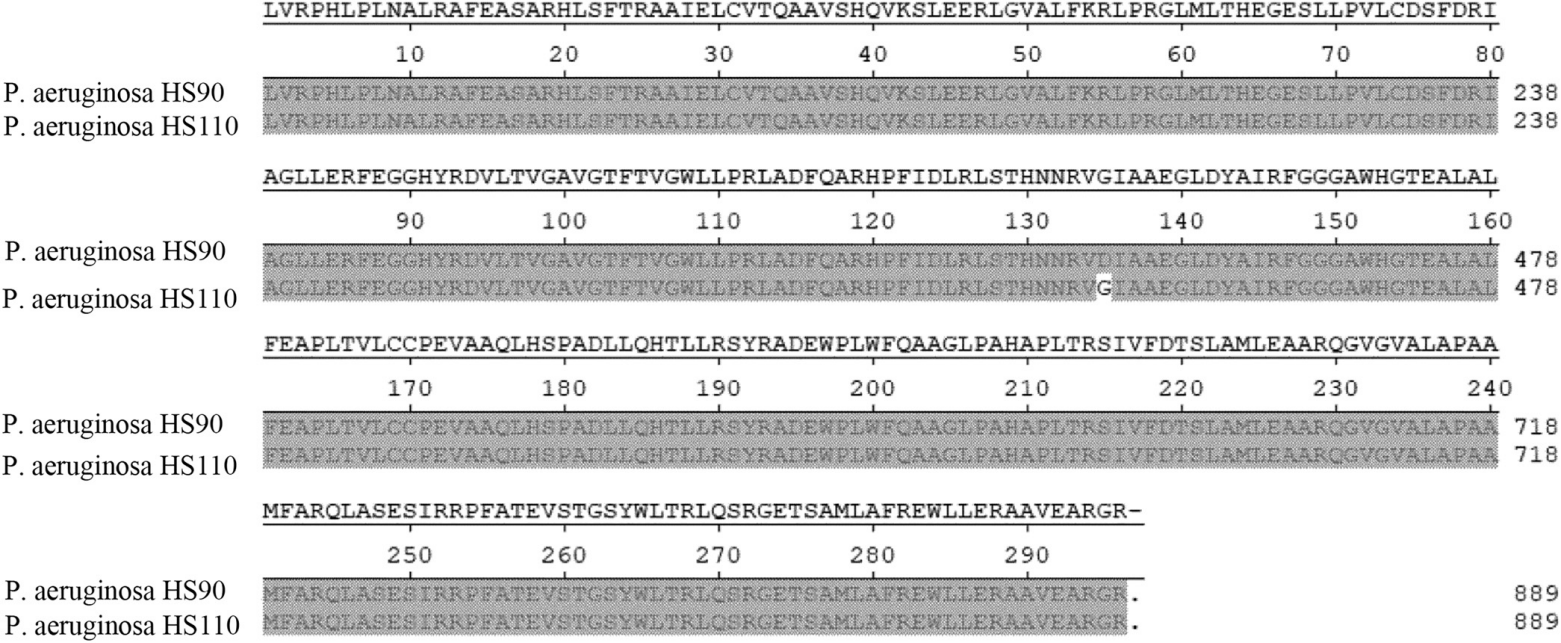
Difference in the ampR region between P. aeruginosa HS90 and P. aeruginosa HS110.

To elucidate the genomic relationship and differences between P. aeruginosa HS90 and P. aeruginosa HS110, we determined and compared their relative genes (including *ampR*, *ampD*, *ampG*, *PBP1*, *PBP2*, *PBP3*, and *PBP4*, etc.). The amino acid substitution of AmpR D135G was discovered in P. aeruginosa HS110 by comparing this in P. aeruginosa HS90 (Fig. 3). These mutations were not observed in other relative genes (including *ampD*, *ampG*, *PBP1*, *PBP2*, *PBP3*, and *PBP4*).

## DISCUSSION

Infections caused by metallo-β-lactamase (MBL)-producing *Enterobacterales* and P. aeruginosa are increasingly reported worldwide and are usually associated with high mortality rates (>30%) (15). According to reports, most *bla*_IMP-45_-positive P. aeruginosa currently occurs in China, with minor differences in antibiotics resistance profiles between strains, and are resistant to β-lactams (except for aztreonam), β-lactam-β-lactamase inhibitor combinations (including ceftazidime-avibactam), aminoglycosides, and quinolones (16).

Aztreonam, an old antibiotic strongly limited by the spread of extended-spectrum β-lactamase (ESBL) and AmpC during the last three decades, is gradually back in the public eye because of its inability to be hydrolyzed by MBL (17). Aztreonam is essential in treating infections caused by MBL-producing carbapenem-resistant *Enterobacterales* (CRE) and CRPA (15). Nevertheless, we compared two CRPA strains harboring *bla*_IMP-45_ isolated from a single patient but exhibiting different aztreonam susceptibility. Both isolates, which belonged to ST773 high-risk clone, had the same β-lactam resistance genes (*bla*_PDC-16_, *bla*_IMP-45_, *bla*_OXA-1_, and *bla*_OXA-395_), which means P. aeruginosa HS110 might have been derived from the P. aeruginosa HS90 clinical isolate by gaining aztreonam resistance via mutations associated with aztreonam resistance.

According to the results of reverse transcription (RT)-PCR, the *bla*_PDC-16_ mRNA level in P. aeruginosa HS110 was about 1,500 times higher than that in P. aeruginosa HS90. In addition, HS110 restored aztreonam susceptibility *in vitro* when adding APB, inhibiting AmpC activity. These findings suggested that overexpression of *bla*_PDC-16_ is the primary resistance mechanism to aztreonam in P. aeruginosa HS110. Chromosomal expression of PDC is tightly regulated by the peptidoglycan (PG) recycling pathway and is triggered by the presence of β-lactams (18). Under normal circumstances, the uridine 5′-pyrophosphoryl-*N*-acetylmuramic acid-pentapeptide (UDP-NAM-P5), classified as *AmpC* repressor, binds to *AmpR*, leading to inhibiting *ampC* expression to basal levels. However, when bacteria are exposed to β-lactamase inducers (such as imipenem), large amounts of muropeptides (including *N*-acetylglucosamine-1,6-anhydro-*N*-acetylmuramyl-peptides and 1,6-anhydro-*N*-acetylmuramyl-peptides) are generated and accumulate in the cytoplasm by increasing NagZ and AmpD activities, leading to *AmpR*-mediated induction of *ampC* expression by substituting UDP-NAM-P5 (19, 20). Mutations in *ampD* and inactivation of PBP4 have also been reported in P. aeruginosa and *Enterobacterales* to induce overproduction of AmpC, leading to pathogen resistance to β-lactams (21). A previous study in 2017 suggested that a substitution causes the resistance of CFE-1 to cephalosporins in AmpR. The aspartic acid at position 135 is modified to alanine to allow the constitutive high-level expression (derepression) of *CFE-1* (22). However, we did not detect the mutations in *AmpG*, *AmpD*, *AmpC*, and penicillin-binding protein genes, which meant D135G substitution of AmpR may be the main reason for P. aeruginosa HS110 resistance to aztreonam. Structural changes in AmpR may affect the efficiency of UDP-NAM-P5 to bind to it, thereby deinhibiting AmpC, which needs further research to confirm.

The choice of antimicrobial agents for treating infections caused by MBL-producing P. aeruginosa is limited because MBL-producing P. aeruginosa often plays resistance to a variety of antimicrobial agents (including carbapenems and ceftazidime-avibactam). It is gratifying that several current antimicrobial agents showed good *in vitro* activity against MBL-producing P. aeruginosa, such as cefiderocol and cefepime-zidebactam (23, 24). In this study, the susceptibility of cefiderocol and cefepime-zidebactam were not affected by the expression of *PDC-16*. These new antimicrobial agents may be potential options for treating MBL-producing P. aeruginosa.

### Conclusions.

P. aeruginosa may develop resistance during prolonged therapy with all antimicrobial agents. This study found that the D135G substitution of AmpR resulted in the loss of inhibition and overexpression of its downstream PDC-16, ultimately making the bacteria resistant to aztreonam. This finding re-emphasizes the importance of timely detection of antimicrobial susceptibility of P. aeruginosa during its infections. In addition, since the emergence of MBL-producing P. aeruginosa, the appropriate therapy to treat its infections is unclear, which is a cause for public concern.

## MATERIALS AND METHODS

### Clinical strains.

P. aeruginosa HS90 and P. aeruginosa HS110, isolated from a male outpatient who suffered from an arm infection after an electrical injury, were collected at Huashan hospital (Shanghai, China) on May 24, 2021, and June 4, 2021, respectively. The first isolate P. aeruginosa HS90 displayed resistance to meropenem, ceftazidime, cefepime, piperacillin-tazobactam, ceftazidime-avibactam, and amikacin but was susceptible to aztreonam, imipenem, cefepime-zidebactam, and polymyxin B. In addition, the fosfomycin MIC was 32 mg/liter. After obtaining antimicrobial susceptibility patterns of P. aeruginosa HS90, anti-infective therapy was administered using a regimen of aztreonam (2 g every 8 h) combined with fosfomycin (12 g every day). During that period, fosfomycin was discontinued because of the shortage of fosfomycin. Unfortunately, rapid mutation occurred after 10 days, making the subsequent isolate P. aeruginosa HS110 resistant to aztreonam. In antimicrobial susceptibility testing, P. aeruginosa ATCC 27853 was included for quality control assessment.

### Antimicrobial susceptibility testing and carbapenemase detection.

MICs were determined by the broth microdilution method recommended by the Clinical and Laboratory Standards Institute (CLSI) with CLSI-recommended MIC breakpoints (25). Imipenem, meropenem, ceftazidime, cefepime, aztreonam, levofloxacin, ciprofloxacin, polymyxin B, piperacillin-tazobactam, cefoperazone-sulbactam, cefepime-zidebactam, ceftazidime-avibactam, amikacin, and cefiderocol (Thermo Fisher Scientific Inc., West Sussex, UK) were tested in this study. Cation-Adjusted Mueller-Hinton Broth (ID-CAMHB) was prepared according to the procedure described by the CLSI to assess cefiderocol MICs. Fosfomycin Etest (bioMérieux, France; contains glucose-6-phosphatase) was used to assess fosfomycin MICs because CLSI contraindicates the use of broth microdilution methods to determine the MIC of fosfomycin.

According to the manufacturer’s instructions, the NG test Carba 5 assay (NG Biotech, France) was used to detect carbapenemases carried by clinic strains. Briefly, a 1-μL loopful of bacteria was mixed with five drops of Carba-5 extraction buffer. A total of 100 μL of the mixture was transferred into the Carba-5 cassette after vortexing, and the results were evaluated after incubation at 23 ± 2°C for 15 min (26). 3-Aminophenyl boronic acid (APB) is known to be a potent inhibitor of class C β-lactamases and has been used successfully in detecting the production of plasmid-mediated class C β-lactamases in *Enterobacterales* (27–29). Aztreonam combined-disc tests alone and with 300 μg of APB were performed to compare inhibitory zone diameters.

### Genomic analysis.

Genomic DNAs of two strains were extracted using the TaKaRa MiniBEST bacteria genomic DNA extraction kit v.3.0 (TaKaRa Bio, Japan) and then subjected to whole-genome sequencing using Illumina (Illumina, San Diego, CA, USA) short-read sequencing (150-bp paired-end reads). Multilocus sequence typing (MLST) and antimicrobial resistance gene analysis was performed using ResFinder 4.1 (https://cge.food.dtu.dk/services/ResFinder/).

### Analysis of gene expression.

The relative mRNA levels of β-lactams resistant genes (*bla*_PDC-16_, *bla*_IMP-45_, *bla*_OXA-1_, and *bla*_OXA-395_) and efflux pump genes (including *MexA*, *MexB*, *MexR*, and *OprM*) were determined by real-time-qPCR, according to the referenced method, with some modifications (30). Total RNA was obtained with the TaKaRa MiniBEST Universal RNA extraction kit (TaKaRa, Dalian, China) according to the manufacturer’s instructions. RT-PCR was performed using SYBR Premix *Ex Taq* (TaKaRa, Dalian, China) on an ABI ViiA 7 real-time PCR system (Thermo Fisher Scientific, USA). The reaction parameters were as follows: 50°C for 2 min and 95°C for 10 min, followed by 40 cycles of 95°C for 15 s and 60°C for 34 s; finally 95°C for 15 s, 60°C for 1 min, and 95°C for 15 s. The *rpsL* gene was performed as the endogenous reference gene. All experiments were repeated in triplicate independently.

### Efflux pump inhibitory assays.

The CCCP and PAβN and inhibitory tests were used to detect the effect of efflux pumps on β-lactam resistance of P. aeruginosa HS110, especially aztreonam. Briefly, both CCCP (MedChemExpress, Shanghai, China) and PAβN (MedChemExpress, Shanghai, China) were prepared at 25 mg/liter. The MICs of β-lactams were performed using the broth microdilution method. Bacterial growth in CAMHB containing β-lactams with and without CCCP or PAβN was evaluated in parallel.

### Data availability.

The data used during the current study are available from the corresponding author upon reasonable request. The bacterial genome sequences have been uploaded to NCBI with the following BioProject ID: PRJNA780409. The study protocol was approved by the Institutional Review Board of Huashan Hospital, Fudan University (approval 2018-408).
